# Supplementary material for: Phase Ib study of enzalutamide with venetoclax in patients with metastatic castration-resistant prostate cancer
Source: Cancer Chemother Pharmacol. 2025 Nov 29;95(1):115. doi: 10.1007/s00280-025-04840-2 (PMC12662848; doi:10.1007/s00280-025-04840-2)
Supplement: Supplementary file 1 — Supplementary file1 (PDF 385 KB) [file 280_2025_4840_MOESM1_ESM.pdf]

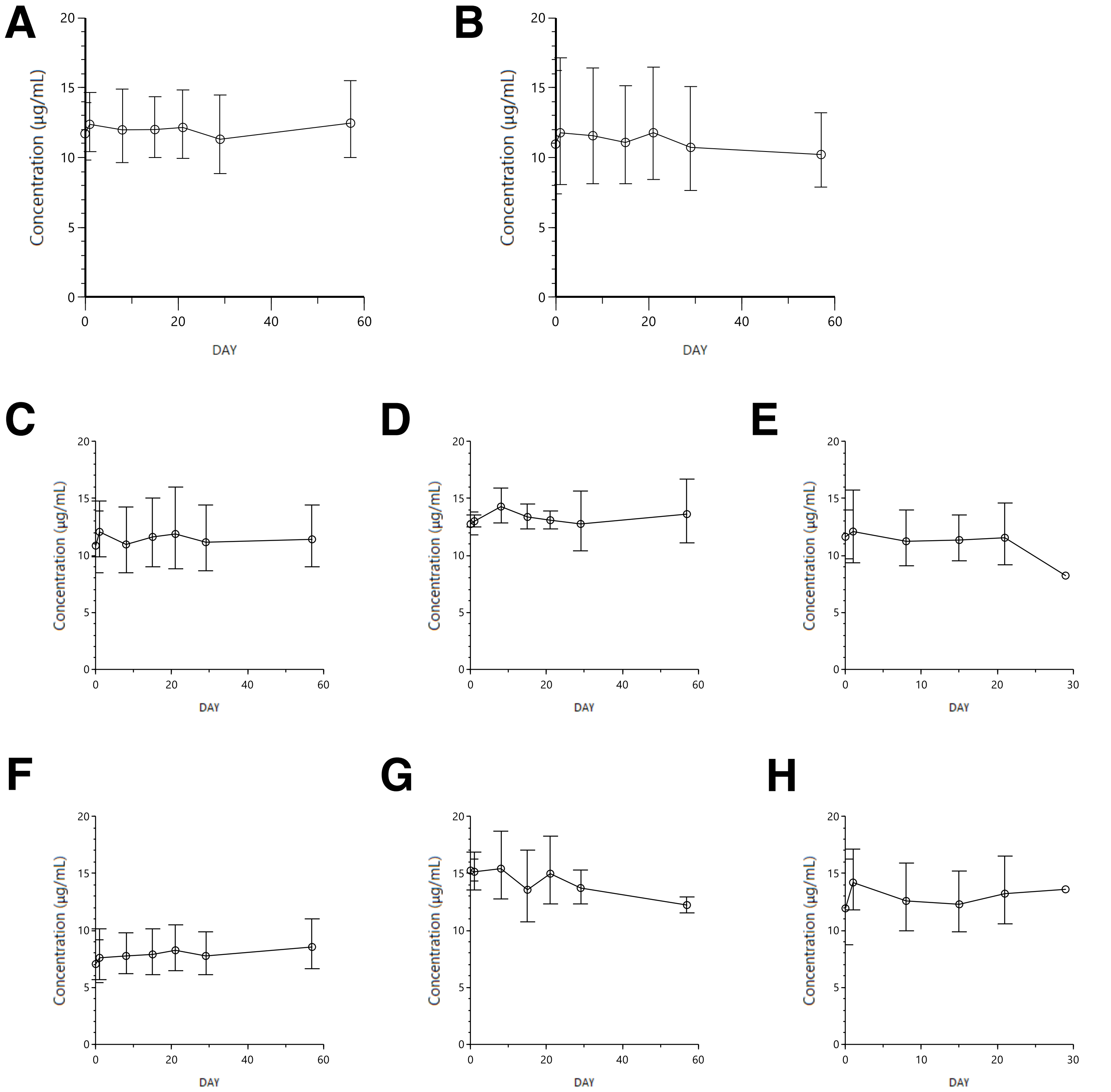

Supplementary Fig. S1: Enzalutamide PK studies.
(**A-B**) Trough enzalutamide (A) and N-desmethyl Enzalutamide (B) concentrations (geometric mean ± geometric standard deviation) over the period of study with venetoclax dosing starting immediately after the day 0 data point.

(C-H) Trough enzalutamide (C-E) and N-desmethyl enzalutamide (F-H) concentrations (geometric mean ± geometric standard deviation) over the period of study with venetoclax dosing starting immediately after the day 0 data point, by dose of venetoclax, 400 mg (C and F), 600 mg (D and G), or 800 mg (E and H).

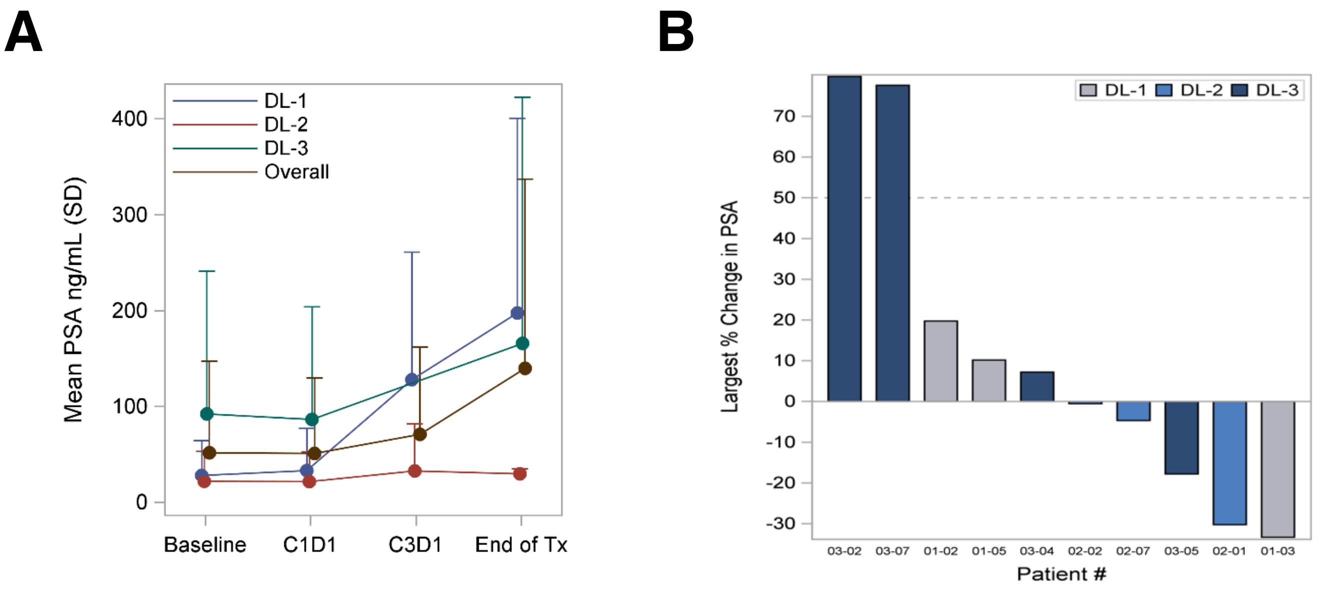

Supplementary Fig. S2. Measures of clinical efficacy of the enzalutamide/venetoclax combination treatment in mCRPC patients (**A-B**) Shown are PSA response summary (A) and waterfall plot (B).

Supplementary Table S1: Patient information (NCT03751436)*

Subject ID	Age (start of treatment)	Age (end of treatment)	Age (when patient passed)	Prior ARPI treatment	Consent	On study	Off treatment	Off study	Deceased/Alive
01-01-RP					ineligible				
**01-02-RP**	59	59	59	Abi x 10 mos	2-Aug-19	9-Aug-19	1-Nov-19	25-Jun-20	DECEASED 6/25/2020
**01-03-RP**	74	77	79	Enza x 2 mos	19-Aug-19	5-Sep-19	15-May-22	15-Aug-22	DECEASED 9/8/2024
01-04-RP					ineligible				
**01-05-RP**	73	73	74	Abi x 4 mos	30-Sep-19	7-Nov-19	28-Jan-20	31-May-21	DECEASED 6/2/2021
**02-01-RP**	67	68	70	No	18-Nov-19	5-Dec-19	5-Aug-20	13-Apr-22	DECEASED 5/5/2022
**02-02-RP**	69	69	70	Enza x 5 mos	30-Dec-19	2-Jan-20	30-Mar-20	3-Jun-21	DECEASED 6/3/2021
02-03-RP					ineligible				
02-04-RP					ineligible				
02-05-RP					ineligible				
02-06-RP					ineligible				
**02-07-RP**	76	77	79	Enza x 10 mos	25-Jun-20	30-Jun-20	15-Mar-21	16-Mar-22	DECEASED 11/2/2022
03-01-RP					ineligible				
**03-02-RP**	63	64	64	Abi x 11 mos	3-Jul-20	7-Jul-20	4-Sep-20	21-Jan-21	DECEASED 1/21/2021
03-03-RP					Withdrawn				
**03-04-RP**	75	75	77	Enza x 7 mos	3-Sep-21	8-Nov-21	5-Dec-21	11-Aug-23	DECEASED 8/11/2023
**03-05-RP**	63	63	x	Enza x 22 mos	12-Oct-21	11-Nov-21	5-Jan-22	5-Jan-23	ALIVE
03-06-RP					ineligible				
**03-07-RP**	74	74	x	Abi x 29 mos	2-May-22	3-May-22	17-Jul-22	18-May-23	ALIVE, still on Pluvicto
03-08-RP					ineligible				

*A total of 20 patients were screened and 10 eligible patients (marked in blue) were enrolled in the Enzalutamide and Venetoclax combination trial, who were put on 3 dose levels (DL) of venetoclax, i.e., DL1 at 400 mg/d (subjects 01-02-RP, 01-03-RP and 01-05-RP), DL2 at 600 mg/d (subjects 02-01-RP, 02-02-RP and 02-07-RP), and DL3 at 800 mg/d (subjects 03-02-RP, 03-04-RP, 03-05-RP and 03-07-RP).

Supplementary Table S2. Enzalutamide and N-desmethyl enzalutamide plasma PK parameters (geometric mean (geometric standard deviation)).

	ENZA		ENZAdm		MR	
Dose (mg)	C_{min} D0[#] (µg/mL)	C_{min} D1[#] (µg/mL)	C_{min} D0[#] (µg/mL)	C_{min} D1[#] (µg/mL)	D0	D1
400 (N=3)	10.8 (1.28)	12.1 (1.23)	7.04 (1.31)	7.59 (1.34)	0.65 (1.08)	0.63 (1.09)
600 (N=3)	12.7 (1.08)	13.0 (1.04)	15.2 (1.07)	15.1 (1.12)	1.20 (1.02)	1.17 (1.16)
800 (N=4)	11.6 (1.20)	12.1 (1.30)	11.9 (1.36)	14.2 (1.21)	1.03 (1.50)	1.17 (1.55)
Total (N=10)	11.7 (1.19)	12.4 (1.19)	11.0 (1.48)	11.8 (1.46)	0.94 (1.43)	0.95 (1.48)
P-value*		0.008		0.652		0.910

[#]Cmin D0 refers to the pre (pre 1st venetoclax) sample on day 1 while Cmin D1 is the 24 h sample relative to Cmin D0.
*Wilcoxon exact sign test (two-tailed, paired), comparing D0 and D1 values for enzalutamide (ENZA), metabolite (ENZAdm), and metabolic ratio (MR), respectively, not corrected for multiple testing.
